# Inflammation’s Association with Metabolic Profiles before and after a Twelve-Week Clinical Trial in Drug-Naïve Patients with Bipolar II Disorder

**DOI:** 10.1371/journal.pone.0066847

**Published:** 2013-06-27

**Authors:** Sheng-Yu Lee, Shiou-Lan Chen, Yun-Hsuan Chang, Po See Chen, San-Yuan Huang, Nian-Sheng Tzeng, Yu-Shan Wang, Liang-Jen Wang, I. Hui Lee, Tzu-Yun Wang, Tzung Lieh Yeh, Yen Kuang Yang, Jau-Shyong Hong, Ru-Band Lu

**Affiliations:** 1 Department of Psychiatry, National Cheng Kung University, Tainan, Taiwan; 2 Institute of Behavioral Medicine, National Cheng Kung University, Tainan, Taiwan; 3 Institute of Allied Health Sciences, College of Medicine and Hospital, National Cheng Kung University, Tainan, Taiwan; 4 Department of Psychiatry, Tri-Service General Hospital, National Defense Medical Center, Taipei, Taiwan; 5 Department of Child and Adolescent Psychiatry, Kaohsiung Chang Gung Memorial Hospital and Chang Gung University College of Medicine, Kaohsiung, Taiwan; 6 Addiction Research Center, National Cheng Kung University, Tainan, Taiwan; 7 Department of Psychiatry, Tainan Hospital, Department of Health, Executive Yuan, Tainan, Taiwan; 8 Laboratory of Toxicology and Pharmacology, NIH/NIEHS, Research Triangle Park, North Carolina, United States of America; Catholic University of Sacred Heart of Rome, Italy

## Abstract

Inflammation is thought to be involved in the pathophysiology of bipolar disorder (BP) and metabolic syndrome. Prior studies evaluated the association between metabolic profiles and cytokines only during certain mood states instead of their changes during treatment. We enrolled drug-naïve patients with BP-II and investigated the correlation between changes in mood symptoms and metabolic indices with changes in plasma cytokine levels after 12 weeks of pharmacological treatment. Drug-naïve patients (n = 117) diagnosed with BP-II according to DSM-IV criteria were recruited. Metabolic profiles (cholesterol, triglyceride, HbA1C, fasting serum glucose, body mass index (BMI) and plasma cytokines (TNF-α, CRP, IL-6, and TGF-β) were measured at baseline and 2, 8, and 12 weeks post-treatment. To adjust within-subject dependence over repeated assessments, multiple linear regressions with generalized estimating equation methods were used. Seventy-six (65.0%) patients completed the intervention. Changes in plasma CRP were significantly associated with changes in BMI (*P* = 1.7E-7) and triglyceride (*P* = 0.005) levels. Changes in plasma TGF-β1 were significantly associated with changes in BMI (*P* = 8.2E-6), cholesterol (*P* = 0.004), and triglyceride (*P* = 0.006) levels. However, changes in plasma TNF-α and IL-6 were not associated with changes in any of the metabolic indices. Changes in Hamilton Depression Rating Scale scores were significantly associated with changes in IL-6 (*P* = 0.003) levels; changes in Young Mania Rating Scale scores were significantly associated with changes in CRP (*P* = 0.006) and TNF-α (*P* = 0.039) levels. Plasma CRP and TGF-β1 levels were positively correlated with several metabolic indices in BP-II after 12 weeks of pharmacological intervention. We also hypothesize that clinical symptoms are correlated with certain cytokines. These new findings might be important evidence that inflammation is the pathophysiology of clinical symptoms and metabolic disturbance in BP-II.

**Trial Registration:**

ClinicalTrials.gov NCT01188148.

## Introduction

Bipolar II disorder (BP-II), defined as recurrent episodes of depression and hypomania, is frequently misdiagnosed in clinical settings [1]–[4]. It is believed that BP-II is greatly under diagnosed in clinical practice and lacks in-depth research because BP-II has been regarded as a “milder form of Bipolar I disorder (BP-I)” [5], [6]. However, long-term follow-ups show that patients with BP-II have a more chronic course, more mood episodes, more major and minor depressive episodes, and shorter inter-episodes, all of which last longer than those of patients with BP-I [7]–[9]. In addition, suicidal risk seems to be particularly elevated in BP-II [10]. The high rate of suicide in BP-II may be explained by frequent misdiagnoses of the disorder followed by ineffective treatment [11], [12].

Inflammation appears to be involved in the pathophysiology, phenomenology, and treatment response of BP [13]. Activation of the inflammatory response system and increased activity of the proinflammatory cytokines interleukin-6 (IL-6), C-reactive protein (CRP), and tumor necrosis factor (TNF-α) were found during acute manic and depressive states [14], [15] [16]–[18]. However, whether these phenomena are state-dependent and normalize in remission remains controversial [19], [20]. These discrepancies may be explained by methodological differences such as heterogeneity in sample characteristics, sample size, medication received, and not controlling for known comorbidities [13]. However, an understanding of an association between inflammation and BP might reveal novel pathophysiology and treatment options, and it might even benefit investigating using anti-inflammatory medications to treat BP [21], [22].

Patients with BP have a higher prevalence of metabolic syndrome than do the general population [23]–[25]. Obesity is more prevalent in BP and is associated with a worse prognosis and with suicide attempts [26]. Several possible factors might contribute to the high risk of metabolic disturbance in patients with BP. Treatment with second-generation antipsychotics and mood stabilizers such as valproate has been associated with altered lipid profiles [27]–[30]. In addition, dysfunction of the HPA axis, dysregulation of hormones, and neurotransmitters in BP might also contribute to metabolic disturbance [31]–[34].

It has been hypothesized that the increased risk for obesity in populations with mood disorder is a consequence of shared pathophysiological pathways. Obesity is proposed as a chronic low-grade proinflammatory state [35]; alterations in CRP and proinflammatory cytokines such as TNF-α and IL-6 are commonly reported in obesity [36]–[38]. Increased plasma transforming growth factor (TGF)-β1, an anti-inflammatory cytokine, was correlated with dyslipidemia in an animal model [39]. Taking these findings together, the bidirectional relationship between metabolic abnormality and BP may be a consequence of aberrant inflammatory networks. Clarifying the correlation between inflammatory markers and the metabolic profile in BP is worth further investigation.

Most studies have evaluated only the association between metabolic disturbance and inflammatory markers during certain mood states instead of evaluating their changes over a prolonged course of treatment. In the present study, we enrolled drug-naïve patients with BP-II and measured their plasma cytokine levels and metabolic indices before and after they had undergone a 12-week pharmacological intervention. We investigated the correlation between changes in inflammatory markers and metabolic indices after 12 weeks of treatment. The correlation between changes in these inflammatory markers and in clinical symptoms was also investigated.

## Methods

### Ethic Statement

The research protocol was approved by the Institutional Review Board for the Protection of Human Subjects at Tri-Service General Hospital and at National Cheng Kung University Hospital. After the study protocol had been completely described to the participants, they all signed written informed consent forms before the trial started and before blood samples were drawn.

### Patient selection

This study is a subgroup analysis of a clinical trial (Trial registration: NCT01188148 at https://register.clinicaltrials.gov/). The protocol for this trial and supporting CONSORT checklist are available as supporting information; see Checklist S1 and Protocol S1. The original study was a randomized, double-blind, controlled 12-week trial, aimed to investigate whether treating bipolar II disorder with valproate (VPA) plus add-on memantine is more effective than VPA alone (Lee et al., unpublished). The original study protocol was to examine 4 subgroups: VPA + placebo, VPA + memantine, VPA + placebo + cognitive behavioral group therapy, VPA + memantine + cognitive behavioral group therapy. However, because of staff shortage, we were unable to perform cognitive behavioral group therapy. Therefore, only the two groups undergoing pharmacotherapy were included in for the entire 12-week study period. Because the aim of the current study was to investigate the association between inflammation and metabolic profiles, we used only patients from the placebo group for this subgroup analysis to avoid the influence of add-on memantine, which is not a routinely used medication in the treatment of bipolar II disorder. In this way, the result would be more applicable to daily practice.

The study population was recruited from outpatient and inpatient settings in Tri-Service General Hospital in Taipei and at National Cheng Kung University Hospital in Tainan, Taiwan. The inclusion criteria were: a) Patients with BP-II initially evaluated in an interview by an attending psychiatrist and followed-up with a more detailed interview with a clinical psychologist using the Chinese Version of the Modified Schedule of Affective Disorder and Schizophrenia-Life Time (SADS-L) [40], which has good inter-rater reliability [41], to determine Diagnostic and Statistical Manual of Mental Disorders (DSM-IV) diagnoses; b)Han Chinese; c) between 18 and 65 years old. The exclusion criteria were: 1) a major mental illness, borderline personality disorder, drug dependence, or a cognitive disorder other than BP-II; 2) previous use of any psychotropic agent; 3) taking any anti-inflammatory medication before or during the trial period.

Although DSM-IV-TR [42] criteria require a minimum duration of 4 days of hypomania, current epidemiologic data samples [2], [7], [43]–[46] suggest that a 2-day duration is more prevalent in the community; therefore, we used the 2-day minimum for hypomania when diagnosing BP-II.

### Study Design

After they had been enrolled in this study, the patients were given open-label valproic acid (500 mg and 1000 mg daily [50–100 μg/ml in plasma]). Only limited use of benzodiazepines (lorazepam; up to 8-mg/day) or fluoxetine (up to 20 mg/day) was allowed as concomitant medication for insomnia, agitation, or irritability. The doses were adjusted based on each patient's clinical manifestations and tolerance. In case of side-effect intolerance or clinical worsening, the patients were withdrawn earlier. The severity of mood symptoms was assessed using the Young Mania Rating Scale (YMRS) [47] and the Hamilton Depression Rating Scale (HDRS) [48], [49]. Clinical ratings were done by research psychiatrists who were trained and experienced in using the rating scales. Symptom severity was assessed at baseline and at 2, 8, and 12 weeks. After the initiation of pharmacological treatment, BMI, lipid profile (cholesterol, triglyceride, high density lipoprotein (HDL), low density lipoprotein (LDL)), fasting serum glucose level, and glycosylated hemoglobin (HbA1C) were measured at baseline and at each visit when symptom severity was assessed.

Ten milliliters of whole blood was withdrawn from the antecubital vein of each patient. Plasma was isolated from the whole blood after it had been centrifuged at 3000 g for 15 min at 4°C, and then it was immediately stored at −80°C. Cytokine levels were quantified using an antibody pair assay system (Flexia; BioSource Intl., Camarillo, CA). Sample processing and data analysis were done according to the manufacturer's instructions. The immunological parameters–CRP, TNF-α, TGF-β, and IL-6–were measured at baseline and at each visit when symptom severity was assessed.

### Statistical Analysis

SPSS 18 for Windows was used for statistical computations. Significance was set at *P*<0.05. Repeated measurements were used to investigate the association of plasma cytokine levels with the metabolic profile (BMI, lipid profile, fasting serum glucose level, and HbA1C) and symptom severity before and after pharmacological treatment. Because all cytokine levels were distributed erratically and showed a significant level of positive skew (Table 1), arithmetic transformations were used to produce approximately normal distributions for further analysis; log (*x*+1) was used for cytokine levels. To evaluate the possible correlations of the plasma cytokines with the metabolic profile (BMI, lipid profile, fasting serum glucose level, and HbA1C) and symptom severity, the multiple linear regression model was used. The statistical method, the generalized estimating equation (GEE) [50], was set up for multiple linear regression in repeated-measures studies which can accommodate randomly missing data [51]. In the current study, GEE analysis was used to investigate the correlations of the plasma cytokines (dependent variables) with the metabolic profile and symptom severity (all parameters of which were independent variables); time effects (treatment period from baseline to week 12), gender and age were controlled for. To adjust for multiple comparisons, a Bonferroni correction for multiple comparisons was done.

**Table 1:** Mean HDRS score, YMRS score, and cytokine and metabolic profiles before and after pharmacological treatment.

	Baseline	After 12 Weeks
Number (n)	117	76
Gender (male/female) (n)	65/52	45/31
Age, mean (SD), (years)	30.66±11.11	30.3±11.1
Age at onset (years)	17.5±5.4	
Number of episodes	4.3±2.6	
HDRS1 score, mean (SD)	19.2±5.4	9.4±6.5
YMRS2score, mean (SD)	9.5±4.6	5.8±3.9
BMI, mean (SD), (kg/m2)	23.4±4.9	24.3±4.7
Glucose AC, mean (SD), (mg/dL)	89.8±17.1	86.4±25.2
Triglyceride, mean (SD), (mg/dL)	92.2±73.3	97.4±64.8
Cholesterol (total), mean (SD),(mg/dL)	179.5±35.1	183.5±35.1
HDL-C, mean (SD), (mg/dL)	58.2±16.0	58.4±16.0
LDL-C, mean (SD), (mg/dL)	109.9±30.4	112.5±30.9
HbA1C, mean (SD), (%)	5.54±0.48	5.61±1.32
Depakine level, mean (SD), (mg/L)		67.5±23.5
CRP (ng/mL)	1906.6±1916.8	1459.5±1630.5
TGF-β1 (pg/mL)	30788.9±16364.5	27145.3±16269.8
TNF-α(pg/mL)	1.74±1.70	1.63±1.80
IL-6 (pg/mL)	1.51±1.75	1.46±2.01

VPA: valproate; HDRS: Hamilton Depression Rating Scale; YMRS: Young Mania Rating Scale; CRP: C-reactive protein; TGF-β1: transforming growth factor β1; TNF-α: tumor necrosis factor α; IL-6: interleukin 6.

The power analysis was done using G-Power 3 software [52], [53], and the effect-size conventions were determined according to Buchner et al. [52].

## Results

The trial ran from 1^st^ August, 2008 to 31^st^ July, 2012. The first patient was recruited on 2009/8/25 while the last enrolled patient finished on 2012/5/30. One hundred seventeen patients were recruited. All patients with BP-II were first diagnosed, and that they had no history of taking mood stabilizers or antipsychotics was verified.

**Figure 1:**
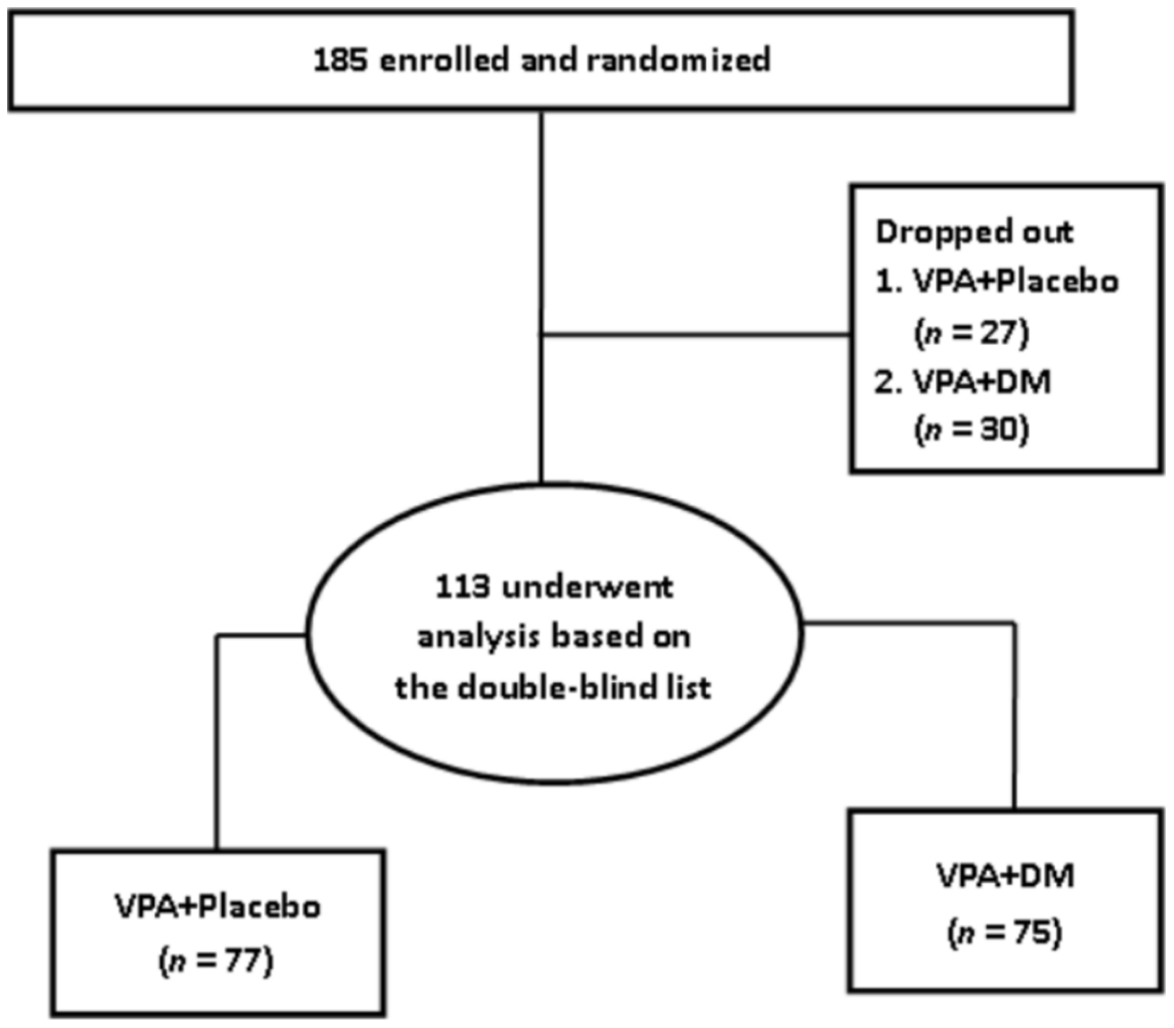
Consort flow chart of recruitment.

At baseline, 117 patients were assessed. One hundred and three patients were assessed when the trial proceeded to week 2. At week 8, eighty patients were assessed. In the end, seventy-six (65.0%) of the 117 patients completed the 12-week pharmacological treatment and metabolic profile survey (Figure 1) The demographic and clinical characteristics, baseline and endpoint HDRS and YMRS scores, metabolic profiles, and plasma cytokine levels of the patients are shown in Table 1.

Correlation analysis was done on all patients in the placebo group. Changes in plasma CRP levels were significantly associated with changes in BMI (*P* = 1.7E-7) and triglyceride (*P* = 0.005) levels (Table 2). However, changes in plasma CRP levels were not associated with changes in cholesterol, HDL, LDL, or HbA1C levels. Changes in plasma TGF-β1 levels were significantly associated with changes in BMI (*P* = 8.2E-6), cholesterol (*P* = 0.004), and triglyceride (*P* = 0.006) levels, but not with the changes in HDL, LDL, or HbA1C levels. Changes in plasma TNF-α and IL-6 levels were not associated with any of the changes in metabolic indices after the 12-week treatment (Table 2). Changes in HDRS scores were significantly associated with changes in IL-6 (*P* = 0.003) levels. In addition, the changes in YMRS scores were significantly associated with changes in CRP (*P* = 0.006) and TNF-α (*P* = 0.039) levels (Table 2). However, if we correct for multiple comparisons, only the associations between CRP and TGF-α1 with BMI (both *P<*0.001) remain significant.

**Table 2:** Correlation of changes in metabolic profiles and cytokines before and after 12 weeks of pharmacological treatment.

	CRP			TGF-β1			IL-6			TNF-α		
	B	95%CI	*P*	B	95%CI	*P*	B	95%CI	*P*	B	95%CI	*P*
HDRS	0.624	−0.28–1.53	0.176	0.062	−1.58–1.70	0.941	2.41	0.81–4.0	0.003	0.027	−1.31–1.37	0.969
YMRS	0.958	0.28–1.64	0.006	−0.47	−1.62–0.67	0.416	0.034	−0.70–0.77	0.929	0.62	0.03–1.21	0.039
Triglyceride	28.03	8.6–47.5	0.005	30.98	8.9–53.0	0.006	14.14	−2.3–30.6	0.092	−0.066	−12.6–12.5	0.992
Cholesterol	−3.571	−9.3–2.2	0.223	13.36	4.3–22.5	0.004	−2.21	−8.0–3.6	0.457	−2.215	−8.3–3.9	0.478
HDL	−4.203	−8.7–0.3	0.067	1.07	−7.5–9.7	0.807	8.73	−0.7–18.1	0.070	−0.511	−3.4–4.4	0.799
LDL	−4.682	−12.1–2.8	0.220	12.4	−2.2–27.0	0.095	0.73	−8.4–9.8	0.876	−4.366	−12.8–4.0	0.308
BMI	3.23	2.0–4.4	1.7E–7**	5.27	3.0–7.6	8.2E–6**	−0.067	−0.3–0.2	0.589	−0.088	−0.92–0.27	0.285
HbA1C	0.032	−0.04–0.11	0.408	0.042	−0.03–0.12	0.283	0.034	−0.02–0.09	0.233	0.024	−0.05–0.95	0.505

CRP: C-reactive protein; TGF-β1: transforming growth factor β1; TNF-α: tumor necrosis factor α; IL-6: interleukin 6.

** ***P***<0.001.

The study had a power of approximately 0.33 to detect a small effect, and 0.99 to detect medium and large effects for multiple regression analysis (*N* = 117). In this power analysis, the effect-size conventions were determined according to Buchner et al. [52] as follows: small effect size  = 0.02, medium effect size  = 0.15, large effect size  = 0.35 for the multiple regression model (alpha  = 0.05).

## Discussion

We found an association between changes in BMI and CRP, one of the markers of low-grade inflammation. This might mean that elevated levels of CRP are also indicators of a relative increase in BMI. The positive association between CRP and BMI is widely known [54]–[57] in healthy controls and other populations (diabetes, hypertension). A significant association between high CRP and prevalence of metabolic syndrome has been reported [58]. Another study [59] also reported that elevated levels of CRP in adulthood are related to changes in BMI between childhood and adulthood [59]. Our findings about the positive association between the longitudinal changes in BMI and CRP support a positive association of BMI and CRP in drug naïve patients with BP-II. In addition, we also report a possible positive association of change in CRP and triglyceride levels which did not survive the correction of multiple comparisons. Whether this finding supports past studies [60]–[62] that suggest the probable involvement of CRP with metabolic syndrome and obesity still requires further study.

CRP is an acute-phase reactant synthesized in the liver largely in response to proinflammatory cytokines. Adipose tissue can produce proinflammatory cytokines that subsequently increase CRP production [63]. CRP does not cross the blood-brain barrier (BBB) in trace amounts; however, during systemic inflammation and obesity, CRP may increase paracellular permeability of the BBB and impair BBB function [64]. In addition, the level of CRP may indicate the status of inflammation in the brain, which might decrease neurotrophic support and lead to brain dysfunction [65]; such dysfunction is also associated with the pathogenesis of BP. Our findings support the hypothesis that inflammation is the common underlying pathogenesis for the frequently comorbid BP and metabolic disturbances.

The current study also provided initial evidence of a positive correlation between TGF-β1 and and BMI. TGF-β1 induces plasminogen activator inhibitor 1 (PAI-1) synthesis and is associated with BMI in humans [66], [67]. However, our findings of the positive correlation between TGF-β and triglycerides and cholesterol did not survive the correction of a multiple comparison. TGF-β1 is a potent anti-inflammatory cytokine that regulates various physiological processes, including cell proliferation, cell differentiation, extracellular matrix synthesis, and the immune response. It has been implicated in the pathogenesis of autoimmune disease, carcinogenesis, and cardiovascular disease [68]–[70]. Evidence indicates that TGF-β is implicated in significantly higher cardiovascular disease plasma levels of activated TGF-β in patients with coronary heart disease [71]. The correlation between hypercholesterolemia and TGF-β1 has been hypothesized to be caused by activation of the innate immune response, inflammation, and fibrosis [39].Our findings confirmed a positive association between BMI and increased plasma TGF-β1in BP-II. Combining the positive association of plasma CRP and TGF-β1 with BMI and possibly dyslipidemia, we propose that the positive association is a result of activation of the immune response, which warrants study of the mechanisms involved.

However, we found no significant association of the metabolic profile with plasma IL-6 and TNF-α levels, as was previously reported [56], [72]. This inconsistency may be explained by different study designs and the study participants in the current study. We analyzed the association of changes in metabolic profiles and inflammatory factors instead of cross-sectional correlations, as was done in previous studies. In addition, all participants in the current study were drug naïve patients with BP-II undergoing initial short-term pharmacological intervention. The long-term metabolic influences and their association with the inflammatory factors in our patients require additionalfollow-up and investigation.

In treating neuropsychiatric disorders, being able to identify and quantify peripheral biomarkers for diagnosis or monitoring treatment responses still remains a clinical goal. We reported that the decreases in HDRS scores over the study period were associated with decreases in IL-6 levels, and that the decreases in YMRS scores were associated with the decreases in CRP and TNF-α levels. However, these findings did not survive correction for multiple comparisons. Based on our findings, we propose that changes the levels of plasma cytokines may be associated with the severity of mood symptoms in patients with BP-II in a prolonged course of treatment instead of in a cross-sectional study. Other studies [15], [17] have suggested that changes in proinflammatory cytokines may be related to the pathophysiology of BP and to the response to pharmacological treatment. A change in IL-6 levels according to mood state is one of the most consistent findings in BP [15], [16]. Our finding supports a report [73] that IL-6 was positively correlated with HDRS scores. In addition, our data suggest that changes plasma CRP may be associated with the severity of manic symptoms, which agrees with Dickerson et al. [14], who said that mania symptoms but not HDRS scores were associated with the increased production of CRP. The possible association between cytokines and clinical symptoms found in the current study requires additional study to confirm. In addition, whether the combination of these cytokines may be used as biomarkers for the severity of BP-II still requires further investigation using a larger sample size.

Our study has some limitations. First, we measured plasma cytokines in the current study because previous studies suggested that changes in peripheral cytokine expression levels may partially reflect the changes in peripheral levels. However, we were unable to draw a definitive conclusion about this hypothesis [74]. Second, except for the correlation between changes in CRP and TNF-α with changes in BMI, most of our positive findings did not survive correction for multiple testing. Further studies with larger samples are needed to verify our results. To achieve enough power (0.8) for detection of small effect (0.02), we would need a target size around N = 395. Third, it is possible that the medication permitted in the study obscured the mood correlation between cytokines and metabolic indices. Although we tried to limit concomitant treatment medication to only three drugs, our results should still be taken with caution. Fourth, the 2-day duration for hypomania used in the current study is not widely accepted. The positive finding in the current study may not apply to patients with BP-II diagnosed according to the DSM-IV-TR criteria of the 4-day duration for hypomania. Fifth, we did not control for the baseline severity of patients. Unlike past studies that focused on only one pole of the mood spectrum, we assessed both the YMRS and HDRS scores for subsyndromal manic symptoms that were presented in over half of bipolar depressive episodes yet frequently not assessed [75]. By evaluating symptoms on both poles of the mood spectrum, our clinical evaluation is more comprehensive, which may better reflect the association of cytokines and the symptoms of BP. Finally, our study focused only on the metabolic influences after short-term pharmacological intervention. The long-term metabolic influences in our patients require additional follow-up and investigation.

In conclusion, our study provides initial evidence of a positive correlation between plasma CRP and TGF-β1 levels and BMI in drug-naïve patients with BP-II after 12 weeks of pharmacological intervention. The specific roles of cytokines in regulating and maintaining peripheral metabolic health require additional investigation. We also hypothesize that clinical symptoms are correlated with certain cytokines. Whether these cytokines can be used as biomarker of BP-II warrants additional studies. We expect that knowledge of the function of plasma cytokines in metabolic disturbances will benefit the development of novel therapies to modify cytokine levels in order to control both clinical symptoms and metabolic disturbances in BP-II.

## Supporting Information

Checklist S1 **CONSORT Checklist.**
(DOC)

Protocol S1 **Trial Protocol.**
(DOC)
