# Supplementary material for: Inflammation’s Association with Metabolic Profiles before and after a Twelve-Week Clinical Trial in Drug-Naïve Patients with Bipolar II Disorder
Source: PLoS One. 2013 Jun 27;8(6):e66847. doi: 10.1371/journal.pone.0066847 (PMC3695222; doi:10.1371/journal.pone.0066847)
Supplement: Protocol S1 — Trial Protocol. (DOC) [file pone.0066847.s002.doc]

**SERIES STUDY OF BIPOLAR DISORDER**

**Specific Aims**

1. We will investigate whether adding on memantine to valproate treatment have synergistic effect and show higher efficacy than each of them in improving symptomatology, anti-inflammatory factors, plasma BDNF, and lipid profile in BP-II patients. The result will help in optimizing the treatment of neuroprotective agents.
2. We will search biological correlates of adding on memantine to valproate treatment response. We predict that patients with altered expressions of inflammatory and neurodegeneration-related genes (e.g. BDNF, TNF-α, …), lower plasma levels of BDNF, lower cytokine levels, more severe baseline depressive symptoms and lipid proflies may be more likely to benefit from such neuroprotective agents.
3. We will compare plasma BDNF, other cytokines, and parameters of lipid profiles before- and after-treatment, and determine whether the genetic variants may be related with the changes of clinical manifestation under memantine add-on treatment of valproate. The results will aid in establishing biological markers for monitoring the course of BP-II and its treatment response.

In summary, we expect that these results will greatly advance the recognition, understanding and clinical application of the neurodegeneration theory of BP-II.

**Background and Rationale**

1. **Clinical Significance of recognition and treatment for BP-II**

Bipolar II disorder (BP-II), defined as recurrent episodes of depression and hypomania, is frequently misdiagnosed in clinical settings (Akiskal & Pinto, 1999; Angst et al., 2003; Benazzi, 2001; Benazzi & Akiskal, 2003). Although BP-II has been perceived as a common disorder with a prevalence of approximately 3–11% (Akiskal 1995; Akiskal &Pinto 1999; Angst *et al*. 2003), 10-15% may eventually die as a result of suicide. Long-term follow-ups show that patients with BP-II have a more chronic course, more mood episodes, more major and minor depressive episodes, and shorter inter-episodes, which last longer than those of patients with BP-I (Judd et al., 2003; Pallanti et al., 1999; Vieta et al., 1997). Psychosocial impairments also significantly increase with each increment in the severity of the symptoms of depression (Judd et al., 2005).

1. **Pathogenesis: bipolar disorder as a neurodegenerative disease**

Neurodegeneration is increasingly recognized as one of the pathogenesis of bipolar disorder (Bauer et al., 2003) supported imaging studies suggesting that ongoing neuronal atrophy accompanies bipolar disorder (Beyer & Krishnan, 2002; Drevets et al., 1997; Stoll et al., 2000). Brain cell dysfunction and possible neuron loss has been proposed to be involved in the pathogenesis of bipolar disorder (Drevets 2000; Peng et al., 2005). Bipolar disorder may be associated with induction of a lot of endotoxins and exotoxins and decrease of neurotrophic factors, which will overactivate microglial cell and inhibit astroglia cell. Those effects may induce pre-inflammatory factors such as TNF-α, C-reactive protein, interleukins, etc. and decrease BDNF etc. which will cause neuron damage or necrosis. The vicious cycle will lead to progressive worsening of the disease.

1. **Treatment for BP-II: Beyond mood stabilizer or antipsychotics**

While the pharmacological guidelines for treatment are well established (American Psychiatric Association 2000; Kowatch *et al*. 2005; Suppes *et al*. 2005; Yatham *et al*. 2005), treatment for bipolar disorder remains less than ideal. Most individuals still have breakthrough episodes or significant residual symptoms while on medication (Bauer *et al*. 2002). In addition, functional deficits often remain even when patients are in remission (Bauer *et al*. 2002). Moreover, most BP-II depression patients are newly diagnosed who have not taken any mood stabilizer or antipsychotics in the past. Because many patients with bipolar disorder remain symptomatic even when fully adherent to their medication regimens, greater understanding of the pathogenesis of this illness and novel treatment modality other than current regimen of mood stabilizers and antipsychotics is needed. The major medication therapy of bipolar disorders is mood stabilizers, However, the pharmacology mechanisms are not clear as yet.

*Mood Stabilizers and Pathogenesis of Bipolar Disorders*

Recent study reported that the common neuroprotective effects of mood stabilizers suggest the role of brain cell dysfunction in bipolar disorder and the dysfunction may eventually develop to neuron loss. Volumetric neuroimaging, now increasingly assessing potential involvement of different brain structures in mood regulation, could be applied to test neuroanatomical models of mood disorders (Strakowski *et al.* 2000). Otherwise, imaging studies suggested that ongoing neuronal atrophy accompanies bipolar disorder For instance, PET images of the cerebral blood flow and the rate of glucose metabolism regarding as brain activity detected the reduced activity in subgenual prefrontal cortex during bipolar depression (Drevets *et al.* 1997). This decrement in activity was, in part, at least explained by a corresponding reduction of cortical volume, as same as magnetic resonance imaging demonstration of the mean grey matter volume. In bipolar disorder, abnormalities of the third ventricle, frontal lobe, cerebellum, and possibly the temporal lobe are also noted (Stoll *et* *al.* 2000; Strakowski *et al.* 2000; Beyer & Krishnan 2002).

1. **Novel treatment model: Memantine as Neuroprotective agents**

***Memantine and neuroprotective effect***

Memantine used to be recognized as a noncompetitive N-methyl-D-aspartate receptor antagonist. It was found to have neuroprotective effect in several neurodegenerative diseases in the recent few years. We found that memantine could inhibit brain inflammatory response through its action on neuroglial cells and provide neurotrophic effect. Mechanistic studies revealed that the high potency of small dosage of memantine is due to its dual actions: an anti-inflammatory effect by reducing the activity of microglia and an increase in the release of neurotrophic factors, such as BDNF, GDNF by astroglia, (Wu *et al*. 2009) (For Hung-Ming Wu, Jau-Shyong Hong, Ru-Band Lu*. US Patent 2009-0118376 A1. May 07, 2009). Our research team also found that even 1/100 dosage of memantine (0.2mg/kg) may be effective in opioid addictive behavior in rat by conditioned place preference (CPP) (filing the patent; Chen et al. 2011). We propose that drugs with neuroprotective effect and neurotrophic effect may treat neurodegenerative diseases including BP-II for memantine, with neuroprotective and neurotrophic effects, may not only inhibit overactivity of microglial cell, but also repair the damaged neurons and neurogenesis through activation of astroglial cell and release of neurotrophic factors.

**Methodology**

This 3-year proposed project will compare the efficacy of add-on memantine and placebo for BP-II depression treatment in a randomized, double-blind, placebo-controlled trial and will explore biological markers for predicting treatment response.

A total of two hundred forty to three hundred twenty BP-II patients will be recruited, from the Department of Psychiatry at National Cheng Kung University Hospital and Tri-Service General Hospital, into this 12-week double-blind, placebo-controlled trial. The research protocol will soon be approved by the institutional review board. After a description of the study to the patients, written informed consent will be obtained.

**Subjects and procedures**

Patients will be evaluated by research psychiatrists after a thorough medical and neurological workup. The Chinese Version of Modified Schedule of Affective Disorder and Schizophrenia-Life Time (SADS-L) (Endicott and Spitzer 1978) will be conducted for confirmation of the diagnosis. Although DSM-IV-TR (American Psychiatric Association 2000) criteria require a minimum duration of 4 days of hypomania, current epidemiologic data suggest that a 2-day duration is more prevalent in community samples (Akiskal et al. 1977, 1979; Angst et al. 2003; Judd et al. 2003); therefore, we used the 2-day minimum for hypomania in the diagnosis of BP-II. Eligible patients, aged 18-55, need to have a DSM-IV-TR (American Psychiatric Association 2000) diagnosis of BP-II. HDRS and YMRS will be used to evaluate severity of mood symptoms. Only Patients with a total of HDRS score at least 18 or YMRS score at least 14 at screen will be recruited. Patients with major, minor mental illnesses other than BP-II will be excluded. Patients who had taken memantine within 1 week before the first dose of the double-blind medication will be excluded as well.

Study procedure: Eligible patients will be randomly assigned into four treatment groups (n = 60-80 for each group) in a double-blind manner and all groups will match in demographic characteristics, symptom severity, while they receive open-label VPA treatment (500 mg and 1000 mg daily [50-100 μg/ml in plasma]) and add-on: 1) memantine (5mg/day), 2) placebo respectively for twelve weeks. Patients will be randomized to receive placebo or any of active treatments in a 1:1 ratio. During the study, only limited use of benzodiazepines (up to 8-mg/day lorazepam), prozac (up to 20mg/day) and risperidone (up to 1-4mg/day) is allowed as concomitant medication for insomnia, agitation or irritable. In case of side-effect intolerance or clinical worsening, the patients will be withdrawn earlier.

**Measures of symptomatology**

Efficacy will be assessed by the YMRS (Young et al. 1978) and HDRS (Hamilton 1960, 1967). Clinical ratings will be performed by research psychiatrists who are trained and experienced in the rating scales. All assessments will be blind to treatment assignment and completed at baseline and the starting day of memantine (1 week after baseline; day 0 of week 1) followed by measurements of treatment responses on day 7 of weeks 1, 2, 4, 8, and 12.Routine laboratory tests, including CBC and biochemistry will be checked at baseline and the end of week 4, 8, and 12.

**Laboratory assessments**

We will measure plasma levels of BDNF, cytokines when treatment response is mearusred. Other parameters of lipid profile will be checked when routine laboratory tests are done.

**Group therapy**

Twelve sessions of group psychotherapy with cognitive techniques will be performed for patients assigned to the VPA+memantine with psychotherapy and the VAP+placebo with psychotherapy groups.

**Statistical analysis**

1. One variable analysis: Explore each subset of demographic characteristics.
2. Two variable analyses: Using Chi-Square, one-way ANOVA, Pearson’s Correlation Coefficient.
3. Multi-variate analyses: Applying multiple regression and logistic regression, analyze each variable (including demographics, symptom severity, plasma BDNF, cytokines, change of lipid profiles and response to medication treatment) in prediction of prognosis in patients with BP-II.
4. The effect size conventions in power analysis will be determined according to Buchner et al. (1996) as follows: small effect size = 0.10, medium effect size = 0.30, large effect size = 0.50 for the test; and small effect size = 0.02, medium effect size = 0.15, large effect size = 0.35 for the multiple regression model (alpha = 0.05).

**Expected Results**

- - 1. We expect that combine therapy of add-on memantine to VPA will exert better efficacy than add-on placebo to VPA in improving symptoms, lipid profile, plasma BDNF and cytokine levels in patients with BP-II.
    2. We expect that alterations of plasma BDNF and cytokines may be related with the changes of clinical manifestation under add-on memantine to VPA treatment and serve as a biomarker for BP-II. By clarifying the cytokine and BDNF profiles of BP, we may more fully understand the pathogenesis of BP.
    3. Ameliorate the paucity of studies of the treatment of BP-II beyond antispsychotics and mood stabilizer.
    4. BP-II is commonly under-recognized and misdiagnosed leading to inappropriate treatment or missed opportunities of treatment which likely to play a part in the increased risk for suicide, mania, and chronic psychosocial suffering in BP-II patients.

In summary, we expect that these results will greatly advance the recognition, understanding, and clinical application of the neurodegeneration theory of BP-II.

**Study flow**

In this 3-year proposal we will enroll 240-360 BP-II patients. Therefore, 80-110 patients will be recruited per year during the first two and half years (30 months). Since this is a 12-week study, the last subject will complete the trial on month 3 of the third year. Laboratory procedures will be performed all over the 3-year study period and completed within the last 3 months. Data analysis and report (and manuscript) preparation will be completed within the last 2 months.

References:

1. Akiskal HS, Djenderedjian AM, Rosenthal RH, Khani MK. Cyclothymic disorder: validating criteria for inclusion in the bipolar affective group. Am J Psychiatry 1977; 134:1227-1233.
2. Akiskal HS, Rosenthal RH, Rosenthal TL, Kashgarian M, Khani MK, Puzantian VR. Differentiation of primary affective illness from situational, symptomatic, and secondary depressions. Arch Gen Psychiatry 1979; 36:635-643.
3. Akiskal, HS, Pinto, O. The evolving bipolar spectrum. Prototypes I, II, III, and IV. Psychiatr Clin North Am 1999; 22:517-534, vii.
4. American Psychiatric Association. Diagnostic and Statistical Manual of Mental Disorders. fourth ed. (DSM-IV). Washington, DC: American Psychiatric Association, 2000.
5. American Psychiatric Association. Practice Guideline for the Treatment of Patients with Bipolar Disorder (revision). Am J Psychiatry 2002; 159(Suppl):1-50.
6. Angst J, Gamma A, Sellaro R, Lavori PW, Zhang H. Recurrence of bipolar disorders and major depression. A life-long perspective. Eur Arch Psychiatry Clin Neurosci, 2003; 253:236-240.
7. Angst J. The bipolar spectrum. Br J Psychiatry 2007; 190:189-191.
8. Angst J. The emerging epidemiology of hypomania and bipolar II disorder. J Affect Disord 1998; 50:143-151.
9. Bauer M, Alda M, Priller J, Young LT. International Group For The Study Of Lithium Treated Patients (IGSLI). Implications of the neuroprotective effects of lithium for the treatment of bipolar and neurodegenerative disorders. Pharmacopsychiatry 2003; 36:S250-254.
10. Bauer M, Unützer J, Pincus HA, Lawson WB; NIMH Affective Disorders Workgroup. [Bipolar disorder.](http://www.ncbi.nlm.nih.gov/pubmed/12558008) Ment Health Serv Res 2002; 4:225-229.
11. Benazzi F, Akiskal, HS. Refining the evaluation of bipolar II: beyond the strict SCID-CV guidelines for hypomania. J Affect Disord 2003; 73:33-38.
12. Benazzi F. Is 4 days the minimum duration of hypomania in bipolar II disorder? Eur Arch Psychiatry Clin Neurosci 2001; 251:32-34.
13. Beyer JL. Krishnan KR. Volumetric brain imaging findings in mood disorders. Bipolar Disorders 2002; 4:89-104.
14. Craddock N, Jones I. Genetics of bipolar disorder. J Med Genet 1999; 36:585-594.
15. Drevets WC, Price JL, Simpson JR Jr, et al. Subgenual prefrontal cortex abnormalities in mood disorders. Nature 1997; 386: 824-827.
16. Drevets WC. Neuroimaging studies of mood disorders. Biological Psychiatry 2000; 48: 813-829.
17. Endicott J, Spitzer RL. A diagnostic interview: the schedule for affective disorders and schizophrenia. Arch Gen Psychiatry 1978; 35:837-844.
18. Hamilton M. A rating scale for depression. J Neuro Neurosurg Psychiatry 1960; 23:56-62.
19. Hamilton M. Development of a rating scale for primary depressive illness. Br J Soc Clin Psychol 1967; 6:278-296.
20. Judd LL, Akiskal HS, Schettler PJ, Coryell W, Maser J, Rice JA, et al. The comparative clinical phenotype and long term longitudinal episode course of bipolar I and II: a clinical spectrum or distinct disorders? J Affect Disord 2003; 73:19-32.
21. Judd LL, Akiskal HS, Schettler PJ, Endicott J, Leon AC, Solomon DA, Coryell W, Maser JD, Keller MB. Psychosocial disability in the course of bipolar I and II disorders: a prospective, comparative, longitudinal study. Arch Gen Psychiatry 2005; 62:1322-1330.
22. Kelsoe JR. Arguments for the genetic basis of the bipolar spectrum. J Affect Disord 2003; 73:183-197.
23. Kowatch RA, Fristad M, Birmaher B, Wagner KD, Findling RL, Hellander M. Child Psychiatric Workgroup on Bipolar Disorder. Treatment guidelines for children and adolescents with bipolar disorder. Journal of the American Academy of Child & Adolescent Psychiatry 2005; 44:213-235.
24. Liu B, Hong JS. Primary rat mesencephalic neuron-glia, neuron-enriched, microglia-enriched, and astroglia-enriched cultures. Methods in Molecular Medicine 2003; 79:387-395.
25. Pallanti S, Quercioli L, Pazzagli A, Rossi A, Dell'Osso L, Pini S, et al**.** Awareness of illness and subjective experience of cognitive complaints in patients with bipolar I and bipolar II disorder. Am J Psychiatry 1999;156: 1094-1096.
26. Peng GS, Li G, Tzeng NS, Chen PS, Chuang DM, Hsu YD, Yang S, Hong JS. Valproate pretreatment protects dopaminergic neurons from LPS-induced neurotoxicity in rat primary midbrain cultures: role of microglia. Brain Research. Molecular Brain Research. 2005; 134:162-169.
27. Stoll AL, Renshaw PF, Yurgelun-Todd DA, Cohen BM. Neuroimaging in bipolar disorder: what have we learned? Biological Psychiatry 2000; 48:505-517.
28. Suppes T, Dennehy EB, Hirschfeld RM, Altshuler LL, Bowden CL, Calabrese JR, Crismon ML, Ketter TA, Sachs GS, Swann AC. Texas Consensus Conference Panel on Medication Treatment of Bipolar Disorder. The Texas implementation of medication algorithms: update to the algorithms for treatment of bipolar I disorder. Journal of Clinical Psychiatry 2005; 66:870-886.
29. Vieta E, Gasto C, Otero A, Nieto E, Vallejo J. Differential features between bipolar I and bipolar II disorder. Compr Psychiatry 1997; 38:98-101.
30. Vieta E, Suppes T. Bipolar II disorder: arguments for and against a distinct diagnostic entity. Bipolar Disord 2008; 10:163-178.
31. Wu HM, Tzeng NS, Qian L, Chen HL, Li Y, Zhang C, Hong JS, Lu RB. Memantine protects dopaminergic neurons against LPS-induced neurotoxicity by inhibiting activation of microglial NADPH oxidase. Neuropsychopharmacology 2009; 34: 2344-2357.
32. Wu YS, Ou CS, Chen HC, Lu RB, Angst J. Validation of the Chinese version of the Hypomania Checklist (HCL-32) as an instrument for detecting hypo(mania) in patients with mood disorders. Journal of Affective Disorders 2008; 106:133-143.
33. Yatham LN, Goldstein JM, Vieta E, Bowden CL, Grunze H, Post RM, et al. Atypical antipsychotics in bipolar depression: potential mechanisms of action. J Clin Psychiatry 2005; 66(Suppl 5):40-48.
34. Young RC, Biggs JT, Ziegler VE, Meyer DA. A rating scale for mania: reliability, validity and sensitivity. British Journal of Psychiatry 1978; 133:429-35.
35. Zhang W, Qin L, Wang T, Wei SJ, Gao HM, Liu J, Wilson B, Liu B, Zhang W, Kim HC, Hong JS. 3-hydroxymorphinan is neurotrophic to dopaminergic neurons and is also neuroprotective against LPSinduced neurotoxicity. FASEB Journal 2005; 19:395-397.
36. Zhang W, Wang T, Qin L, Gao HM, Wilson B, Ali SF, Zhang W, Hong JS, Liu B. Neuroprotective effect of dextromethorphan in the MPTP Parkinson's disease model: role of NADPH oxidase. FASEB Journal 2004; 18:589-591.
